# Association between vitamin D concentration and delirium in hospitalized patients: A meta-analysis

**DOI:** 10.1371/journal.pone.0281313

**Published:** 2023-02-08

**Authors:** Ningning Fu, Mengrong Miao, Ningning Li, Shuang Zeng, Ruilou Zhu, Jiaqiang Zhang

**Affiliations:** Department of Anesthesia and Perioperative Medicine, Henan Provincial People’s Hospital, Zhengzhou University People’s Hospital, Zhengzhou, Henan Province, China; Universita degli Studi dell’Insubria, ITALY

## Abstract

**Background:**

Now the occurrence of delirium is more concerning to clinicians and psychiatrists. It has been reported that vitamin D deficiency may be a relevant factor in the development of delirium in hospitalized patients.

**Study objective:**

To investigate the association between vitamin D concentration and delirium in hospitalized patients.

**Design:**

Meta-analysis.

**Methods:**

A systematic literature search was conducted using PubMed, EMBASE, and the Cochrane Library. The primary outcome was the occurrence of delirium in the inpatient setting. Odds ratios (OR) were calculated with random or fixed effects models.

**Results:**

In this article, we define the normal range of vitamin D concentrations as greater than 75 nmol / L, 50–75 nmol / L as vitamin D insufficiency, 25–50 nmol / L as vitamin D deficiency, and less than 25 nmol / L as vitamin D severe deficiency. The Results showed that severe vitamin D deficiency (OR: 1.98 [1.41–2.79], P<0.001) and vitamin D deficiency (OR: 1.50 [1.12–2.00], P = 0.006) were more likely to develop delirium than normal vitamin D levels. Subgroup analysis also revealed that low vitamin D concentrations were associated with a higher incidence of delirium, whether the cutoff point was 25 nmol/L (OR: 1.52 [1.40–1.64], P<0.001), 50 nmol/L (OR: 1.47 [1.19–1.82], P<0.001), or 75 nmol/L (OR: 1.54 [1.21–1.96], P<0.001). The included studies scored medium and high on the Newcastle-Ottawa quality assessment scale.

**Conclusion:**

Compared with normal vitamin D levels, severe vitamin D deficiency and vitamin D deficiency, but not vitamin D insufficiency, are associated with a higher incidence of delirium in hospitalized patients.

**Trial registration:**

This review was registered in the PROSPERO database under identifier CRD42021271347. https://www.crd.york.ac.uk/prospero/display_record.php?ID=CRD42021271347.

## 1. Introduction

Delirium, occurs in hospitalized patients more often than in the elderly. It mainly shows disorders of consciousness, disorganized thinking, lack of attention, emotional disorders, etc., and is often accompanied by other symptoms, and severe cases are even life-threatening [1]. The pathophysiological causes of delirium remain unclear but are generally thought to be related to acute infections, drugs, general anesthesia, surgery, endocrine disorders, cardiovascular disease, and several other factors (hypoxic damage to the brain, poisoning) [2]. Available data suggest that deficiencies of omega-3 polyunsaturated fatty acids and B vitamins cause cardiovascular and neurological disorders, including paresthesias, memory loss, and dementia, among others [3,4]. Omega-3 polyunsaturated fatty acids, like vitamins and minerals, are essential for the human body and have anti-inflammatory and vasodilatory properties, which may protect blood vessels and maintain blood perfusion in the brain. Meanwhile, there is increasing evidence suggesting that vitamin D deficiency may have an association with delirium [5,6].

Vitamin D is a fat-soluble vitamin that has many physiological functions in the human body, such as promoting bone growth, regulating calcium and phosphorus metabolism, regulating immune function, and so on [7]. Several studies have also confirmed the potential role of vitamin D in neurological and neuropsychiatric disorders, and vitamin D may be an important regulator of brain development [8,9]. In addition to this, there is increasing evidence that the vitamin D metabolic pathway may play an important role in obstetric and gynaecological diseases and reproduction, such as endometriosis, ovarian cancer, polycystic ovary syndrome (PCOS), and even breast cancer [10,11]. There are currently no specific guidelines for vitamin D supplementation in affected women, but there should be selectivity in the decision to supplement vitamin D, as its excess may have deleterious effects on fertility [10].

25 hydroxyvitamin D (25 (OH) D) is the intermediate form of vitamin D that is converted in the liver, and it is generally considered as a marker to assess whether vitamin D is deficient [12]. According to the American Endocrine Society guidelines, a serum 25 (OH) D concentration of 25 nmol/L was defined as severe deficiency, a serum 25 (OH) D concentration of 25–50 nmol/L as deficiency, a serum 25 (OH) D concentration of 50–75 nmol/Las insufficiency, and a serum 25 (OH) D concentration of > 75 nmol/L as normal [13,14]. Its role in calcium homeostasis and bone has been extensively studied. Of course, there are also some studies showing some effects of vitamin D on cognition and delirium [5,15,16].

A review by Groves et al summarizes the association of vitamin D deficiency in adulthood with brain related adverse outcomes such as neuropsychiatric disorders and neurodegenerative diseases [17]. Qiu et al reported that preoperative vitamin D deficiency was closely related to postoperative delirium [14]. Ford et al. found that vitamin D levels are lower in patients with delirium compared to those without delirium [18]. Pilling et al. support that low levels of vitamin D are a risk factor for the delirium [19]. However, Morandi et al. concluded that low concentrations of vitamin D levels were not correlated with whether delirium developed and that 25 (OH) D levels in patients with delirium were not significantly different from those in healthy patients [20]. Several studies also did not find an association between low levels of vitamin D and cognitive decline [15,21,22]. To date, whether serum vitamin D deficiency is a risk factor for delirium has not reached a consensus, and no meta-analysis of these issues has been performed. A meta-analysis is urgently needed to resolve these controversies. Thus, we conducted a meta-analysis to investigate the association between vitamin D concentration and inpatient delirium. The primary outcome of this meta-analysis was the association between low levels of vitamin D and the risk of delirium in the inpatient setting, and the other outcomes were the relationships between other potential risk factors and the risk of delirium. We hypothesized that low vitamin D concentrations are a risk factor for delirium in hospitalized patients.

## 2. Methods

The current meta-analysis aims to investigate the relationship between inpatients’ delirium and low levels of vitamin D. Our systematic review was registered before the start of the literature search under the PROSPERO registration number CRD42021271347. This meta-analysis was conducted following the requirements of the MOOSE statement [23].

### 2.1. Eligibility criteria

Inclusive criteria: 1. population: patients with serum 25(OH)D levels detected; 2. intervention vs comparator: low levels of vitamin D vs high levels of vitamin D; 3. the outcomes were the risk of delirium in patients with low levels of vitamin D and patients with higher levels of vitamin D. Represents in the form of OR, HR, etc., or enough data to calculate OR; 4. study design: observational study.

Exclusion criteria: 1. those studies that could not provide enough data to calculate the incidence of delirium in vitamin D deficient patients. 2. animal studies, reviews, case reports, meeting abstracts and meta-analyses, etc.

### 2.2. Information sources

Without language restrictions, we will search the following literature databases: PubMed, EMBASE and the Cochrane Library. All the literature until September 2021 will be searched. References in the included studies were also evaluated.

### 2.3. Search strategy

The keywords vitamin D, 25(OH)D, VitD, 25-hydroxyvitamin D, 25OHD, vitaminD2, vitamin D3, Hydroxycholecalciferols, hypovitaminosis D, and delirium were retrieved using medical subject headings (mesh) and text words combined. **S1 Table** details the complete search strategy for PubMed.

### 2.4. Study selection

Titles and abstracts of articles were independently screened by MM and NL. Full text searches were performed for eligible articles. Any discordant opinions were discussed by all authors until consensus was reached.

### 2.5. Data extraction

Two authors extracted relevant information after screening the literature according to the inclusion and exclusion criteria. Author, year of publication, study type, the population included in the study, OR the value of delirium in different levels of vitamin D concentration, etc. The above data was extracted into an Excel sheet. If studies provided vitamin D concentrations of ng/ml, we performed data transformation by the formula: 1 ng/ml = 2.5 nmol/L VitD.

### 2.6. Methodological quality

Each enrolled article was assessed by two independent personnel using the Newcastle Ottawa scale (NOS) [12]. This scale contains study population selection, study method comparability comparison, exposure, or outcome assessment with 3-part 8 entries for a total of 9 points. The total score was 0–3 points for low-quality literature, 3–6 points for moderate-quality literature, and 6–9 points for high-quality literature. If the two investigators disagreed in the assessment of literature quality, an agreement was reached after seeking a third individual for discussion.

### 2.7. Statistical analysis

Statistical analysis was performed using Stata 14.0 software. To evaluate the relationship between the degree and concentration of vitamin D deficiency in patients with delirium and healthy controls, raw data from each included article was extracted **S2 Table**. Effect sizes for dichotomous outcomes were assessed with odds ratios (OR) and 95% confidence intervals (CI). Continuous variable outcomes were presented with the weighted mean difference (WMD) and associated 95% CI. *I*^*2*^ value to assess the heterogeneity of the included literature, when *I*^*2*^ was less than 50%, the possibility of heterogeneity among studies was low, using the fixed effect model; Otherwise, a random effects model was used. Subgroup analysis based on possible heterogeneity factors, the robustness of results, detectable with sensitive assays.

## 3. Results

### 3.1. Study selection and characteristics

**Fig 1:**
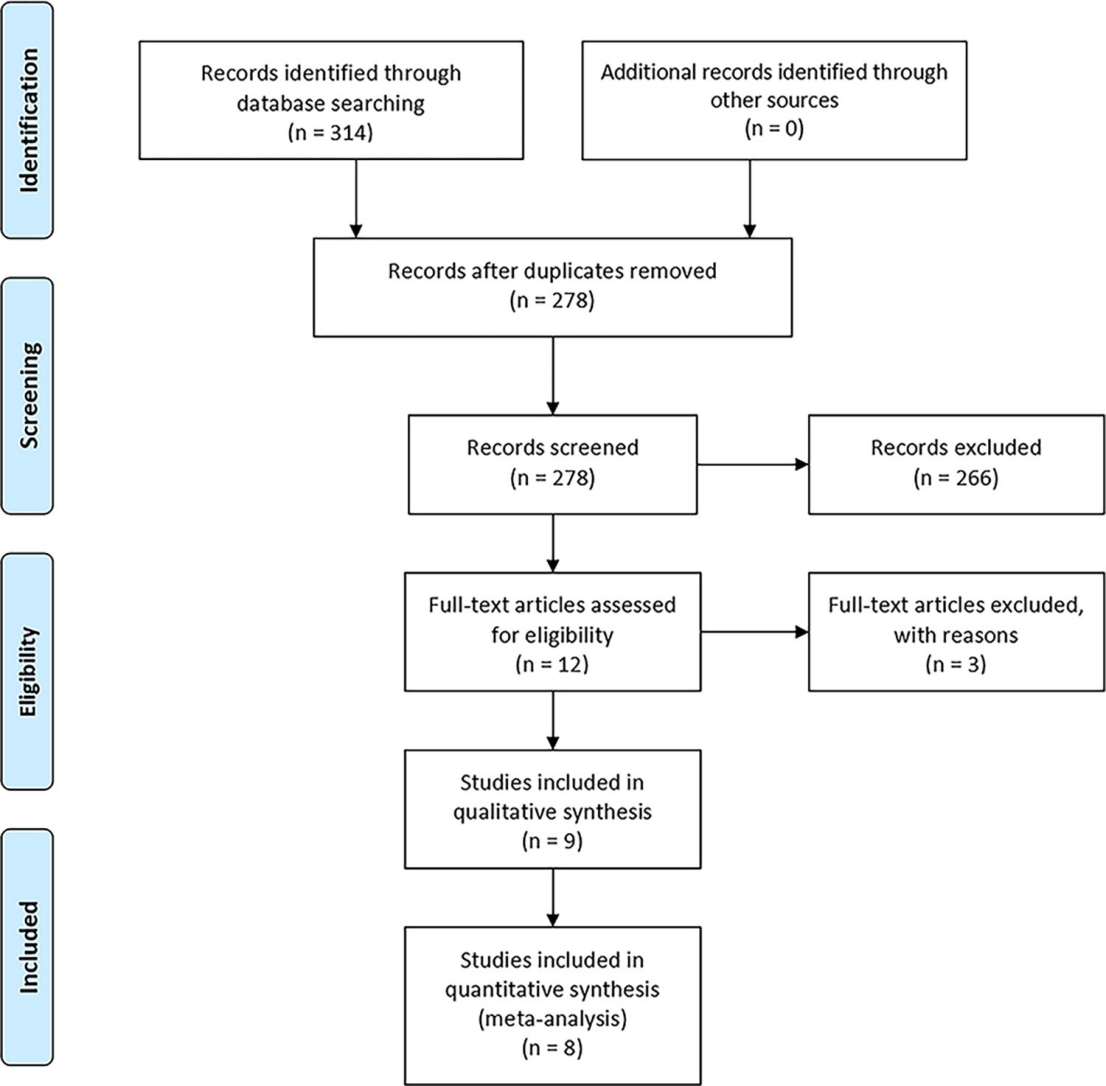
Flow diagram of study screening and selection.

According to the comprehensive search of the above databases, a total of 314 pieces of literature were screened. After checking to remove duplicates, 278 articles remained. Then, based on the inclusion and exclusion criteria, filter the title and abstract, and finally, select 12 articles. 3 articles [6,18,24] were excluded after a full-text search, 9 observational studies did the quantitative analysis. One [25] was excluded because it only provided relevant OR values, the outcome measures did not correspond, and attempts to contact the authors did not obtain the data. Finally, 8 pieces of literature [14,19,20,26–30] were included in the quantitative synthesis in the meta-analysis **(Fig 1)**. For the final quantitative analysis, eight observational studies (three prospective, four retrospective, and one case-control study) were included. The years of publication of these observational studies were between 2013 and 2021. In one [20] of them, there is no detailed data in the literature, so only OR values were extracted.

### 3.2. Quality of evidence

The NOS scale was used to assess the quality of non-RCT studies, with a total of eight entries and nine assigned points (from 0 to 9 stars). The basic characteristics and NOS scores of the included articles are presented in **Tables 1 and 2**. After repeated evaluation by multiple researchers, the average score of NOS is 7.8, which can be considered as high-quality literature.

**Table 1:** The basic characteristics of the included articles.

Study ID	Population	Studytype	Patients	Exposure	Measure Type	Outcome	Diagnostic criteria
Morandi A(2013) [20]	American	Prospective cohort	120 patients aged 40–62 years	Measurement of serum 25(OH)D concentration at the time of study enrollment	chemiluminescence immunoassay	Delirium during critical illness	CAM-ICU
Quraishi SA(2015) [30]	American	Retrospective cohort study	The mean age of 4508 patients was 59, and the standard deviation was 18	Pre-admission serum 25(OH)D level	radioimmunoassay	Hospital-acquired new-onset delirium	The new presence of ICD-9-CM codes related to delirium
Pilling LC(2021) [19]	England	Prospective cohort analysis	351,320 participants who reached age 60 before the end of the follow-up period	Serum 25(OH)D levels at baseline	chemiluminescence	Hospital-diagnosed delirium	International Diagnosis of Diseases, Tenth Revision (ICD-10) code F05
Tumer NB(2020) [28]	turkey	Retrospective analysis	212 adult patients above 65 years of age	Preoperative Vitamin D Level	NA	Postoperative delirium after cardiac surgery	CAM-ICU
Velayati A(2020) [27]	Iran	Prospective Cohort Study	The mean age of 398 patients was 60.97 and the standard deviation was 9.29	Serum levels of 25(OH)D were measured upon admission	NA	Postoperative delirium after coronary artery bypass grafting	CAM-ICU
Qiu Y(2021) [14]	American	Retrospective analysis	632 adult patients	The most recent serum 25(OH) D concentration between 7 and 365 days before surgery	NA	Postoperative delirium in critically ill patients	CAM-ICU
Chouët, Justine(2020) [29]	France	A case control	240 patients aged 70 years and older consecutively	Serum concentrations of 25(OH)D were measured at the time of the CAM test	radioimmunoassay	Delirium among acute geriatric inpatients	CAM
Ingstad, F(2021) [26]	Norway	Retrospective analysis	872 patients aged 40–104 years	Serum concentrations of 25(OH)D were measured a the first or second postoperative day.	liquid chromatography–tandem mass spectrometry	Postoperative delirium after hip fracture surgery	NA
Torbergsen, A C(2015) [25]	Norway	A case control	115 elderly patients	Serum levels of 25(OH)D were measured upon admission	radioimmunoassay	Postoperative delirium after hip fracture surgery	CAM

Abbreviations CAM-ICU: Confusion Assessment Method for the ICU NA: not available.

**Table 2:** Quality assessment of cohort studies using the Newcastle Ottawa scale.

Reference	Selection				Comparability		Outcome			Score
	Representativeness of the exposed cohort	Selection of the non exposed cohort	Ascertainment of exposure	Demonstration that outcome of interest was not present at start of study	Age	Additional factors	Assessment of outcome	Was follow-up long enough for outcomes to occur	Adequacy of follow up of cohorts	
Morandi A(2013) [20]	*	*	*		*	*	*	*		7
Quraishi SA(2015) [30]	*	*	*	*	*	*	*	*		8
Pilling LC(2021) [19]	*	*	*	*	*	*	*	*	*	9
Tumer NB(2020) [28]	*	*	*	*	*	*	*	*		8
Velayati A(2020) [27]	*	*	*	*	*	*	*	*	*	9
Qiu Y (2021) [14]	*	*	*	*	*	*	*	*		8
Ingstad, F(2021) [26]	*	*	*		*	*		*		6
**Reference**	**Selection**				**Comparability**		**Exposure**			**Score**
	case definition adequate	Representativeness of the cases	Selection of Controls	Definition of Controls	Age	Additional factors	Ascertainment of exposure	Same method of ascertainment for cases and controls	Non-Response rate	
Chouët, Justine(2020) [29]	*	*	*	*	*	*	*	*		8
Torbergsen, A C(2015) [25]	*	*	*	*		*	*	*		7

A high-quality selection was considered a star. The more stars assigned to a study, the better the quality of the study. The "comparability" category receives a maximum of two stars. In the “selection” and "outcome/ exposure" categories, each numbered item can receive a maximum of one star.

### 3.3. Evaluation of publication bias and sensitivity analysis

**Fig 2:**
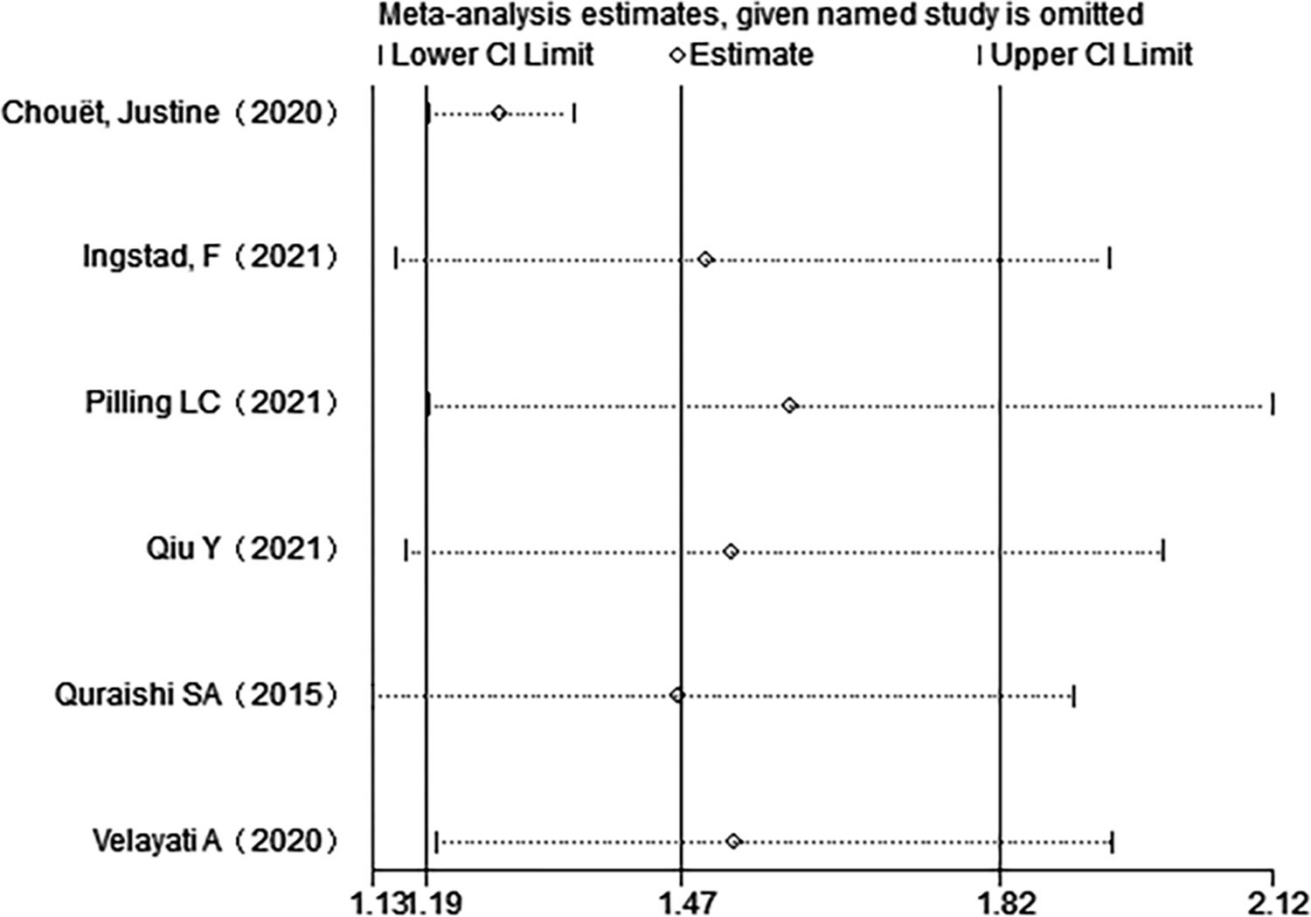
Sensitivity analysis when 50 nmol /L was used as the cutoff.

No publication bias was done in this paper due to the small number of included works of literature. When heterogeneity was high, a sensitivity analysis was performed. After excluding the literature by article, the results were consistent before and after the outcome measures, which indicated that the results were robust and credible (**Fig 2**).

### 3.4. Association between different levels of vitamin D concentrations and the occurrence of delirium in hospitalized patients

**Fig 3:**
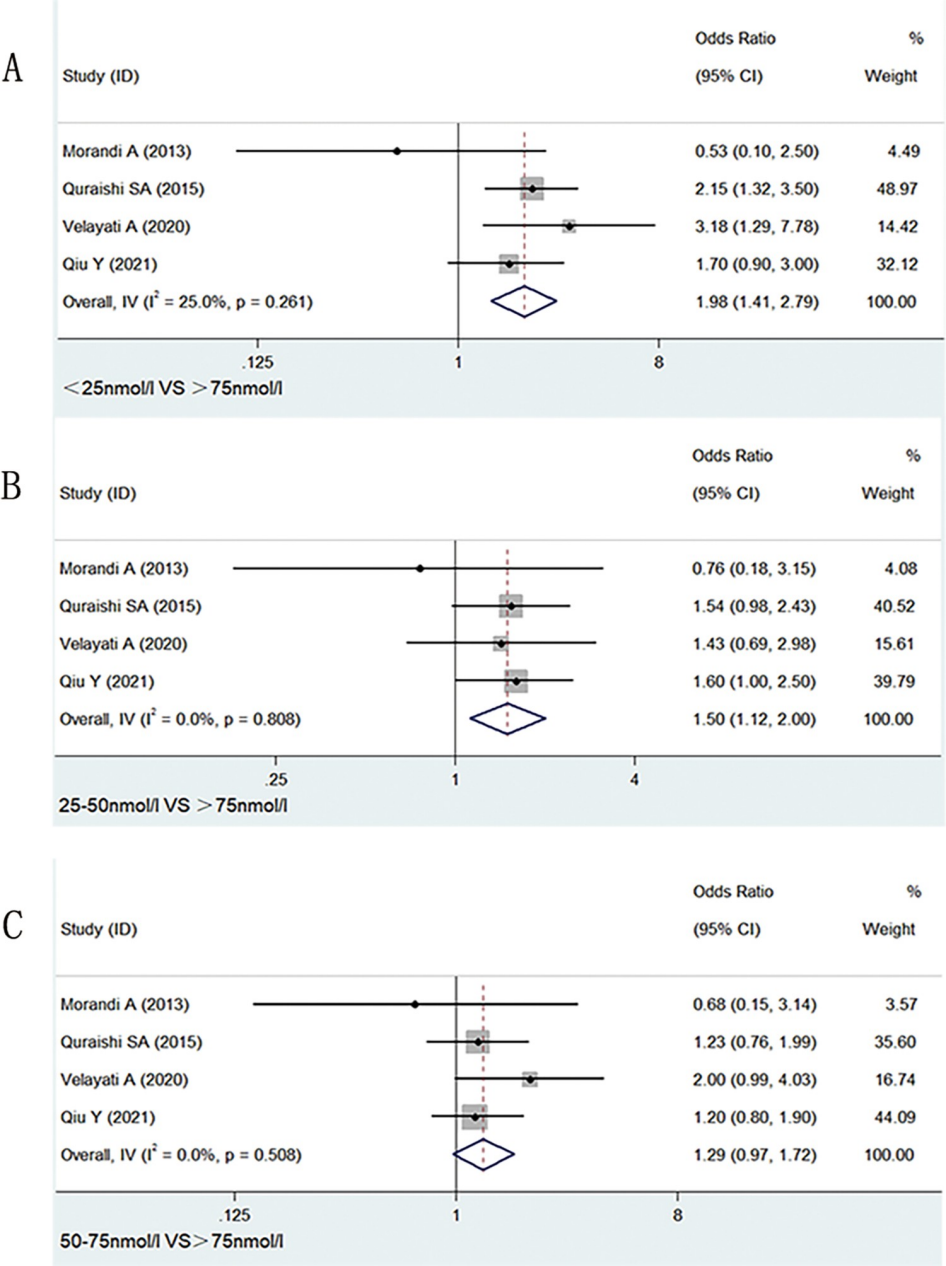
Meta-analysis of the incidence of delirium with low versus normal vitamin D concentrations(>75nmol/L). A: <25nmol/L. B: 25-50nmol/L. C: 50-75nmol/L.

As shown in **Fig 3**, the incidence of delirium was higher in patients with severe deficiency of serum vitamin D than in those with normal vitamin D levels (OR: 1.98; 95% CI: 1.41 to 2.79; *I*^*2*^ *=* 25%, P<0.001). When serum vitamin D was deficient, the incidence of delirium was also greatly different between cases and controls (OR: 1.50; 95% CI: 1.12 to 2.00; *I*^*2*^ *=* 0.0%, P = 0.006). However, when serum vitamin D insufficiency (50–75 nmol/L) was compared with normal serum vitamin D (> 75 nmol/L), there was not statistical difference between the two groups (OR: 1.29; 95% CI: 0.97 to 1.72; *I*^*2*^ *=* 0.0%, P = 0.08). Heterogeneity *I*^*2*^ of less than 50% was observed at the time of meta-analysis, so we adopted a fixed-effects model.

### 3.5. Association with delirium at different threshold concentrations of vitamin D in cases and controls

**Fig 4:**
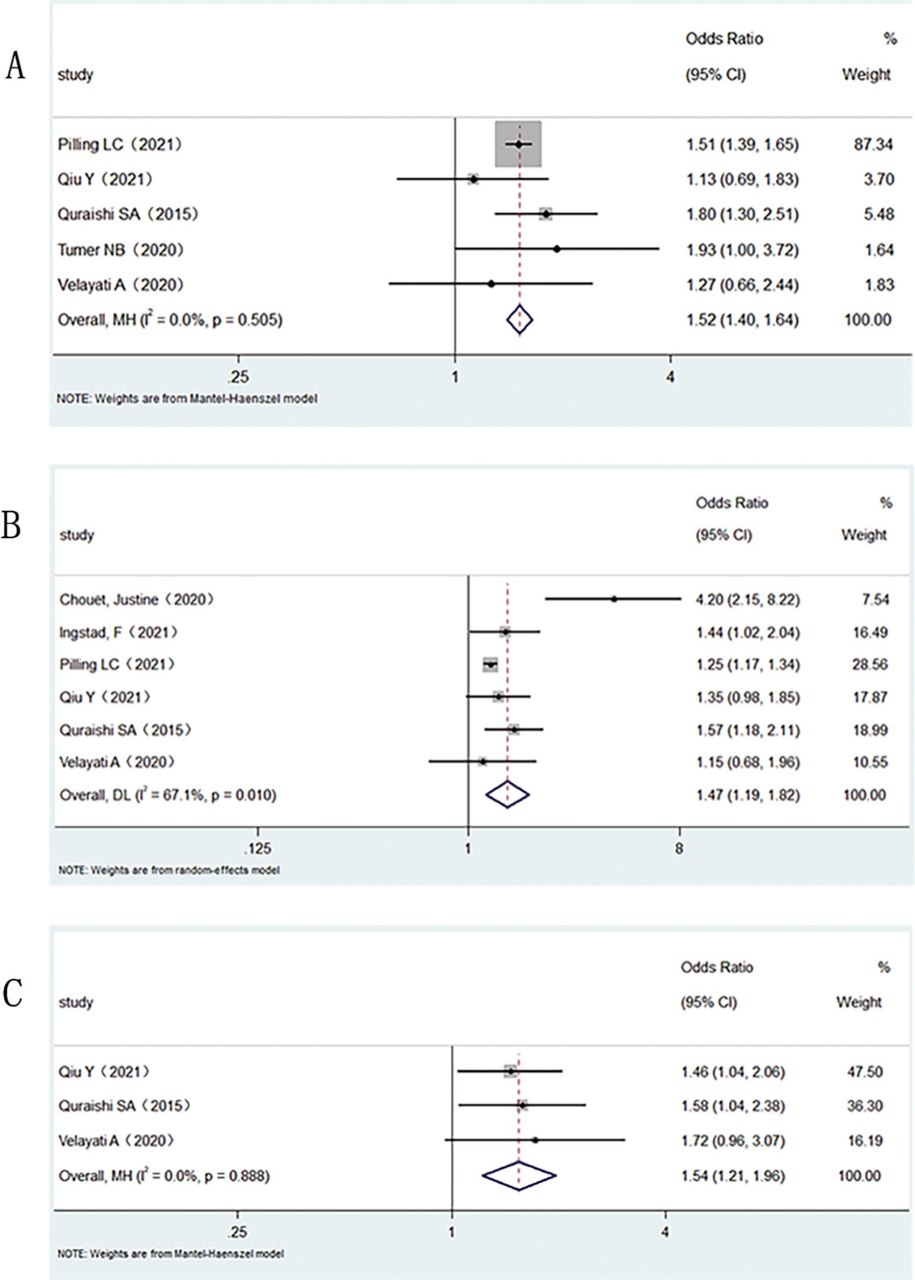
Meta-analysis of the incidence of delirium with different threshold concentrations of vitamin D in cases and controls. A: 25nmol/L. B: 50nmol/L. C: 75nmol/L.

As can be seen in **Fig 4**, Five studies compared the incidence of delirium using a cut-off value of 25 nmol/L and showed a marked difference between groups for serum vitamin D 25 nmol/L (OR: 1.52; 95%CI: 1.40–1.64; *I*^*2*^ *=* 0%, P<0.001). Six observational studies reported the incidence of delirium at less than 50 nmol/L. The results of the meta-analysis showed a striking difference between the groups (OR: 1.47; 95%CI: 1.19–1.82; *I*^*2*^ *=* 67%, P<0.001). As *I*^*2*^ was greater than 50% (*I*^*2*^ *=* 67%), the random effects model was chosen to reduce heterogeneity. Three articles were included when 75 nmol/L was taken as the threshold. The results of the meta-analysis showed that there was still a striking difference in the incidence of delirium when the concentration of serum vitamin D was less than 75 nmol/L (OR: 1.54; 95%CI: 1.21–1.96; *I*^*2*^ *=* 0%, P<0.001).

### 3.6. Subgroup analysis

When we took the 50 nmol / L as the cutoff value for statistical analysis, a large statistical heterogeneity was found (*I*^*2*^ = 67%). This may be due to the use of different assessment tools for delirium in the included studies, type of inpatient population, clinical setting, etc. Through subgroup analysis, different types of hospitalized patients, types of included studies, and the age difference between groups all revealed that low level of vitamin D was a risk factor for the occurrence of delirium. (**Table 3**). Subgroup analyses for other relevant study characteristics are detailed in **Table 3.**

**Table 3:** Subgroup analysis of outcomes.

Subgroup	Trials	N	I^2^ (%)	OR (95% CI)	P value
**Patient type**					
** Postoperative population**	3	1902	0	1.35(1.09–1.67)	0.006
** Others**	3	356068	86.2	1.77(1.13–2.77)	0.01
**Study type**					
** Retrospective cohort study**	3	6012	0	1.46(1.22–1.75)	P<0.001
** Prospective cohort analysis**	2	351718	0	1.25(1.17–1.33)	P<0.001
**Age**					
** No significant difference between groups**	3	5620	75.3	1.93(1.20–3.09)	0.006
** Significant differences between groups**	3	356226	16.3	1.26(1.18–1.35)	P<0.001
**Delirium assessment tools**					
** CAM-ICU**	2	1030	0	1.29(0.99–1.70)	0.06
** Others**	4	356940	79.9	1.63(1.18–2.23)	0.003
**Methods for 25(OH)D quantification**					
** Radioimmunoassay**	2	4748	85.6	2.45(0.94–6.39)	0.07
** Others**	4	353222	0	1.26(1.18–1.34)	P<0.001
**Clinical settings**					
** ICUs**	3	1270	80.8	1.78(0.93–3.41)	0.08
** Combination of hospital wards and ICUs**	3	356700	28.9	1.27(1.19–1.35)	P<0.001

### 3.7. Other potential risk factors for delirium

Analysis of risk factors for delirium showed that neither antipsychotic use, female gender, smoker, serum albumin content, nor BMI were risk factors for delirium in the inpatient setting (**Table 4**). However, a meta-analysis of age showed that patients with delirium were older (WMD: 4.14; 95% CI: 0.34–7.93; *I*^*2*^ = 89.7%, P = 0.03)

**Table 4:** Potential risk factors for delirium in hospitalized patients.

Prognostic factor	Trials	N	I2 (%)	OR/WMD (95% CI)	P value
**Antipsychotic medications**	3	4863	96.4	3.45(0.68–17.55)	0.14
**Female gender**	4	5261	0	0.90(0.71–1.13)	0.37
**Smoker**	2	513	0	0.66(0.38–1.13)	0.13
**Albumin**	2	355	89.7	-0.02(-3.94–3.80)	0.99
**BMI**	2	513	0	-0.5(-1.45–0.44)	0.30
**Age**	4	5261	89.7	4.14(0.34–7.93)	0.03
**Length of hospital stay (days)**	2	610	77.7	1.53(0.55–2.51)	0.002

### 3.8. Length of hospital stay

Length of hospital stay was reported in 2 trials. Meta analysis showed that the length of hospital stay(WMD: 1.53; 95% CI: 0.55–2.51; *I*^*2*^ = 77.7%, P = 0.002) was significantly different between the two groups (**Table 4**).

## 4. Discussion

In recent years, many studies have evaluated the relationship between low-level vitamin D concentrations and delirium in hospitalized patients, but the results are still controversial. To pool and quantitatively evaluate the current evidence, we conducted a meta-analysis of eight studies. The main research conclusions of this paper: 1. Vitamin D deficiency and severe vitamin D deficiency are risk factors for delirium in hospitalized patients.2. There was no significant difference in the incidence of delirium between vitamin D insufficiency and normal vitamin D concentrations. According to the selection of the research population included in the article, the comparability between groups and the evaluation of results in statistical analysis, and the overall quality of evidence, varies from medium to high.

The involvement of vitamin D in various brain and neurocognitive processes has been supported by an increasing number of studies. Two meta-analyses on the relationship between Alzheimer’s disease and vitamin D concentrations also suggested that lower vitamin D concentrations were associated with a higher risk of Alzheimer’s disease [31,32]. To investigate the causal relationship between vitamin D and Alzheimer’s disease, Mendelian randomization studies [33–36] have shown an inverse causal relationship between vitamin D gene levels and Alzheimer’s disease. One [34] study confirms that genetic vitamin D levels can reduce the age threshold for risk of Alzheimer’s disease in people aged 60 years and older. Interestingly, there is one Mendelian randomization study [37] showed no association between genetic risk scores and cognitive performance for SNPs in the DHCR7 and cyp2r1 gene regions. This does not conflict with previous results, as the subjects studied were those with cognitive abilities rather than Alzheimer’s disease. The results of a meta-analysis by Goodwell et al. [16] found that low vitamin D levels were associated with poor cognitive function. Delirium and cognitive function are two mutually independent concepts whose association relationship has not been fully elucidated, but they are both behavioral manifestations of brain dysfunction. Our results showed that lower levels of vitamin D are a risk factor for delirium in hospitalized patients.

The occurrence of delirium in hospitalized patients causes great concern among physicians and patients. The mechanisms underlying the development of delirium remain poorly defined, but increasing inflammation may be an important risk factor for the development of delirium [38,39]. Regarding the contribution of neuroinflammatory pathways to promoting delirium occurrence may be relevant in elderly subjects or patients with previous dementia [39,40]. There is compelling evidence that the presence of peripheral immune signals ultimately leads to functional and structural changes in brain parenchymal cells, including microglia, astrocytes, and neurons, through a complex communication system involving the blood-brain barrier [39]. These neuroinflammatory changes are associated with acute onset of cognitive, behavioral, and mood disorders.

Several possible mechanisms for the emergence of the above results may be 1. The occurrence of delirium at low levels of vitamin D may be related to the pro-inflammatory properties of vitamin D [20]. There is one study showing that when vitamin D is deficient, intake of vitamin D can reduce the C-reactive protein ratio and reduce inflammation [41]. This may be related to the immunomodulatory (related to T cells, B cells, dendritic cells and macrophages) effects of vitamin D [42]. 2. It is already known that the vitamin D receptor can be found in nearly all regions of the brain (cortex, hippocampus, and hypothalamus, among others). Vitamin D can upregulate the expression of vitamin D receptors, thereby having neuroprotective effects against glutamate toxicity and reducing the production of oxidative stress [5]. 3. Vitamin D can influence the genetic expression of delirium-related neurotransmitters such as acetylcholine, serotonin, dopamine, and aminobutyric acid within the neural brain [5]. From the genetic level, Bowman et al. validated the relationship between vitamin D levels and delirium, showing that correcting low levels of vitamin D is necessary to prevent the occurrence of delirium [6].

Currently, opinions remain inconsistent regarding the definition of the adequacy of vitamin D levels. The UK National Institute for Health and Care Excellence considers serum 25 (OH) D levels above 50 nmol/L to be adequate [19]. The Endocrine Society’s clinical practice guidelines recommend that serum 25(OH) D concentrations greater than 75 nmol/L be considered normal [14]. Therefore, we use different cut-off points for analysis. The results showed that low vitamin D concentrations were strongly associated with the delirium in hospitalized patients, whether defined by a cut-off value of 25 nmol/L, 50 nmol/L, or 75 nmol/L. However, the study also found that vitamin D insufficiency did not increase the incidence of delirium relative to normal vitamin D. Taken together, 75 nmol/L may not be the cut-off value to judge vitamin D supplementation’s effectiveness in preventing delirium. A previous study showed that vitamin D concentrations of > 75 nmol/L are necessary for good cognitive function [43]. Our results are not consistent with the above recommendations. It may be that our subjects were patients diagnosed with delirium; it may also be that the meta-analysis included only 4 observational studies with insufficient statistical power. More randomized trial studies are needed in the future to verify this view.

However, the present study also has some limitations. First, the quality of the literature was not high; and the results were underpowered. Second, due to the wide variation in the timing of vitamin D measurement, type of inpatient, and outcome reporting across the included studies, considering the differences and the small number of included articles, we did not perform subgroup analyses. 25 hydroxyvitamin D (25 (OH) D) is the best indicator to reflect vitamin D status in humans, but large inter assay variation in results affects the clinical diagnosis of vitamin D deficiency. Currently available options are the accuracy-based vitamin D plans (ABVD) and treatments from the United Kingdom’s Vitamin D External Quality Assessment Scheme (DEQAS) and the U.S. College of American Patholo-gists(CAP) [44]. Finally, serum levels of vitamin D change dynamically during hospitalization due to various factors such as inflammatory changes, intravenous fluids, and so on. We did not evaluate the delirium effect of vitamin D over time due to limited evidence, so the conclusions should be interpreted with caution. With further enrichment of relevant literature, future meta-analyses may include this issue.

Our study is the first meta-analysis describing the relationship between vitamin D and delirium, and the results showed that vitamin D severe deficiency and vitamin D deficiency are risk factors for delirium, suggesting that it is necessary to detect serum vitamin D levels in hospitalized patients and evaluate the need for supplementation with vitamin D preparations. Healthy men seem to use 3000–5000 IU of cholecalciferol per day, which can meet more than 80% of their winter cholecalciferol requirements [45]. The recommended intake of vitamin D is now insufficient to maintain serum concentrations of 25 (OH) d in the absence of substantial amounts of vitamin D. The results of a meta-analysis [46] suggest that rapid normalization of vitamin D in the setting of acute critical illness is best achieved using a loading dose approach, with cholecalciferol of 10000 IU / kg (maximum 400000 IU) recommended most appropriately based on age or weight. The most recent review, which analyzed the efficacy and safety of calcifediol and cholecalciferol drugs in the short—and long-term, found that calcifediol was more effective with no increased toxicity [47]. A recent study also showed that rapidly increasing vitamin D levels can enhance the body’s immunity against infection [48]. This may provide a way for clinicians and psychiatrists to think about the prevention and treatment of delirium.

## 5. Conclusion

In conclusion, our meta-analysis of observational studies suggests that vitamin D severe deficiency and vitamin D deficiency can increase the incidence of delirium in hospitalized patients. In addition, the study also found that 75 nmol/L may not be the threshold for the need for vitamin D supplementation preparations. The studies we included were observational, and the number of pieces of literature was small. More high-quality randomized trial studies are therefore needed to confirm this.

## Supporting information

S1 Table The complete search strategy of PubMed. (DOCX)

S2 Table Original data of included articles. (DOCX)
