# Supplementary material for: Association between vitamin D concentration and delirium in hospitalized patients: A meta-analysis
Source: PLoS One. 2023 Feb 8;18(2):e0281313. doi: 10.1371/journal.pone.0281313 (PMC9907811; doi:10.1371/journal.pone.0281313)
Supplement: S1 Table — (DOCX) [file pone.0281313.s001.docx]

**S1 Table.** **The complete search strategy of PubMed**

| Number | Search terms |
| --- | --- |
| 1 | "Vitamin D"[Mesh] |
| 2  3  4  5  6  7  8  9  10  11  12  13 | vitamin D  25OHD  25(OH)D  VitD  vitamin D2  vitamin D3  25-hydroxyvitamin D  Hydroxycholecalciferols  hy-povitaminosis D  1 OR 2 OR 3 OR 4 OR 5 OR 6 OR 7 OR 9 OR 9 OR 10  "Delirium"[Mesh]  Delirium |
| 14 | Subacute Delirium |
| 15 | Delirium, Subacute |
| 16 | Deliriums, Subacute |
| 17 | Subacute Deliriums |
| 18  19  20  21  22 | Delirium of Mixed Origin  Mixed Origin Delirium  Mixed Origin Deliriums  12 OR 13 OR 14 OR 15 OR 16 OR 17 OR 18 OR 19 OR 20  11 AND 21 |
