# Supplementary material for: Association between vitamin D concentration and delirium in hospitalized patients: A meta-analysis
Source: PLoS One. 2023 Feb 8;18(2):e0281313. doi: 10.1371/journal.pone.0281313 (PMC9907811; doi:10.1371/journal.pone.0281313)
Supplement: S2 Table — (DOCX) [file pone.0281313.s002.docx]

**S2 Table. Original data of included articles**

| study ID | ＜25nmol/l | | 25-50nmol/l | | 50-75nmol/l | | ＞75nmol/l | | ＜50nmol/l | | ≥25nmol/l | | ≥50nmol/l | | Relative effect (95% CI) |
| --- | --- | --- | --- | --- | --- | --- | --- | --- | --- | --- | --- | --- | --- | --- | --- |
|  | N delirium | N | N delirium | N | N delirium | N | N delirium | N | N delirium | N | N delirium | N | N delirium | N |  |
| Morandi A(2013) |  |  |  |  |  |  |  |  |  |  |  |  |  |  | OR(vs ＞75nmol/l): ＜25nmol/l 0.53(0.1-2.5) 25-50nmol 0.76(0.18-3.15) 50-75nmol/l 0.68(0.15-3.14) |
| Quraishi SA(2015) | 51 | 742 | 71 | 1542 | 50 | 1342 | 27 | 882 |  |  |  |  |  |  | OR(vs ＞75nmol/l): ＜25nmol/l 2.15(1.32-3.50) 25-50nmol 1.54(0.98-2.43) 50-75nmol/l 1.23(0.76-1.99) |
| Pilling LC(2021) | 629 | 42916 | 1521 | 145890 |  |  |  |  |  |  |  |  | 1484 | 162514 | HR(vs ＞50nmol/l): ＜25nmol/l 1.38(1.28-1.49) 25-50nmol 2.49(2.24-2.76) |
| Tumer NB(2020) | 48 | 138 |  |  |  |  |  |  |  |  | 16 | 74 |  |  |  |
| Velayati A(2020) | 14 | 70 | 16 | 94 | 20 | 90 | 18 | 144 |  |  |  |  |  |  | OR(vs ＞75nmol/l): ＜25nmol/l 3.18(1.29-7.78) 25-50nmol 1.43(0.69-2.98) 50-75nmol/l 2(0.99-4.03) |
| Qiu Y(2021) | 43 | 75 | 124 | 209 | 88 | 159 | 91 | 189 |  |  |  |  |  |  | OR(vs ＞75nmol/l): ＜25nmol/l 1.7(0.9-3.0) 25-50nmol 1.6(1.0-2.5) 50-75nmol/l 1.2(0.8-1.9) |
| Chouët, Justine(2020) |  |  |  |  |  |  |  |  | 74 | 162 |  |  | 13 | 78 | OR(vs ＞50nmol/l): ≤50nmol 2.37(1.07-5.25) |
| Ingstad, F(2021) |  |  |  |  |  |  |  |  | 84 | 407 |  |  | 71 | 465 | OR(vs ≥50nmol/l): ＜50nmol/l 1.48（1.04-2.12） |
| Torbergsen, A C(2015) |  |  |  |  |  |  |  |  |  |  |  |  |  |  | OR(vs ≥50nmol/l): ＜50nmol/l 2.7 (1.04–7.2) |
